# The effects of water temperature on cerebral blood flow during aquatic exercise

**DOI:** 10.1007/s00421-023-05264-7

**Published:** 2023-07-07

**Authors:** Howard H. Carter, Oliver Pienaar, Alexander Coleman, Jem L. Cheng, Maureen J. MacDonald, Louise H. Naylor, Daniel J. Green

**Affiliations:** 1 School of Sport Science, Exercise and Health, The University of Western Australia, Crawley, WA 6009 Canada; 2 Department of Kinesiology, McMaster University, Hamilton, ON Canada; 3 School of Humans Sciences (M408), 35 Stirling Highway, Crawley, Perth, WA 6009 Canada

**Keywords:** Water immersion, Aquatic exercise, Cerebral blood flow, Thermoregulation

## Abstract

**Purpose:**

Recent studies suggest that episodic increases in cerebral blood flow (CBF) may contribute to the improvement in brain health associated with exercise training. Optimising CBF during exercise may enhance this benefit. Water immersion in ~ 30–32 °C augments CBF at rest and during exercise; however, the impact of water temperature on the CBF response has not been investigated. We hypothesised that cycle ergometry in water would increase CBF compared to land-based exercise, and that warm water would attenuate the CBF benefits.

**Methods:**

Eleven young heathy participants (nine males; 23.8 ± 3.1 yrs) completed 30 min of resistance-matched cycle exercise in three separate conditions; non-immersion (Land), 32 °C and 38 °C water immersion up to the level of the waist. Middle cerebral artery velocity (MCAv), blood pressure, and respiratory measures were assessed throughout the exercise bouts.

**Results:**

Core temperature was significantly higher in the 38 °C immersion than 32 °C (+ 0.84 ± 0.24 vs + 0.04 ± 0.16, *P* < 0.001), whilst mean arterial pressure was lower during 38 °C exercise compared to Land (84 ± 8 vs 100 ± 14 mmHg, *P* < 0.001) and 32 °C (92 ± 9, *P* = 0.03). MCAv was higher in 32 °C immersion compared to the Land and 38 °C conditions throughout the exercise bout (68 ± 10 vs 64 ± 11 vs 62 ± 12 cm/s, *P* = 0.03 and *P* = 0.02, respectively).

**Conclusion:**

Our findings suggest that cycle exercise in warm water attenuates the beneficial impact of water immersion on CBF velocity due to redistribution of blood flow to subserve thermoregulatory demand. Our findings suggest that, whilst water-based exercise can have beneficial effects on cerebrovascular function, water temperature is a key determinant of this benefit.

## Introduction

In humans, repeated episodic increases in blood flow are associated with improvement in arterial health, at least partly due to shear-stress-induced upregulation of endothelial cell function and the bioavailability of paracrine hormones including nitric oxide (NO) (Joyner and Green 2009; Hambrecht et al. 2003; Green et al. 2017). Recent studies indicate that shear stress induces NO-mediated arterial vasodilation in carotid arteries (Hoiland et al. 2021; Carter et al. 2016), whilst animal studies indicate that cerebral vessels are shear-stress sensitive (Fujii et al. 1991; Gaw and Bevan 1993) and that endothelial cells contribute to the beneficial effects of exercise on cerebrovascular function and health (Leblond et al. 2013; Gertz et al. 2006; Endres et al. 2003). Based on these findings, it has been proposed that exercise training may exert beneficial effects on cerebrovascular health via its episodic impacts on cerebral blood flow (CBF), shear stress and endothelial function. Some recent longitudinal training studies support this proposal (Green et al. 2021; Guadagni et al. 2020), highlighting the importance of characterising the CBF response during exercise to assist in optimising cerebrovascular benefits.

Our group (Carter et al. 2014; Pugh et al. 2014), and others (Parfitt et al. 2017; Shoemaker et al. 2019), have previously reported that lower body water immersion induces an increase in CBF velocity, both at rest and during exercise. Water-based exercise, therefore, minimises musculoskeletal risk in frail patients, whilst potentially improving cerebrovascular health. To date, no study has investigated the impact of water temperature on CBF during aquatic exercise, despite the fact that whole body heating and water immersion both impact systemic haemodynamics. The impact of warmer temperatures on CBF has some translational relevance, as previous studies have associated habitual sauna bathing with decreased risks of cardiovascular disease, dementia and Alzheimer’s (Laukkanen et al. 2016, 2018). This highlights the need to characterise CBF in response to a range of conditions to identify the optimal stimulus for enhancement of brain health. We hypothesised that aqua-cycling in 38 °C water would attenuate the increase observed at 32 °C, as blood flow is redistributed to the cutaneous circulation for the dissipation of heat.

## Methods

### Participants

Eleven healthy young participants were recruited (nine males; age 23.8 ± 3.1 yrs; height 180 ± 12 cm; weight 75.0 ± 16.5 kg; BMI 23.0 ± 2.9 kg/m^2^). Exclusion criteria included any history of cardiovascular, cerebrovascular, metabolic and/or respiratory disorders and musculoskeletal injuries, and smokers. Female participants were tested either during the early follicular phase of their natural menstrual cycle (days 1–7 of the cycle) (*n* = 1) or during the placebo pill phase of their hormonal contraceptive cycle (*n* = 1). This study complied with the Declaration of Helsinki and was approved by the Human Research Ethics Committee of the University of Western Australia (Ref: RA/4/1/5642). Participants were provided with a document outlining the experimental procedures, and all participants provided written, informed consent prior to commencing the study. The datasets generated during and/or analysed during the current study are available from the corresponding author on reasonable request.

### Experimental design

In randomised order, and on different days, each participant performed three separate bouts of cycle exercise, including 32 °C and 38 °C water immersion and a non-immersion condition (Land), with a minimum of 48 h between each trial. Trials were conducted at the same time of day (± one hour) to eliminate potential circadian variation effects on vascular function. Participants arrived at the laboratory having fasted for a minimum of 8 h and having abstained from caffeine, vigorous physical exercise and alcohol for a minimum of 24 h. Upon arrival for each session, height, weight, resting blood pressure and core temperature were measured, after which subjects sat and rested for a minimum of 20 min whilst being instrumented for the cerebral measures. Subjects were then placed on a cycle ergometer in a tank which was either empty (Land condition), or filled to the level of the umbilicus in the 32 °C or 38 °C water conditions. Instruments were attached and calibrated, at which point an initial 10-min rest period began. After the 10-min rest period, subjects underwent three 10-min stages of 60 rpm ergometer cycling at increasing resistances: 5 kg, 10 kg and 15 kg, before a final recovery period of 5 min. Resistance was applied via a brake that was in contact with a stainless steel disc wheel built around the pedal hub. All cerebrovascular, respiratory and systemic haemodynamic measures were recorded continuously throughout the exercise bout and averaged over the 5 min prior to each time point. Brachial artery blood flow was assessed for 1 min at the end of every 10-min exercise block. Atmospheric temperature for the Land, 32 °C and 38 °C conditions was 22 ± 2 °C, 24 ± 6 °C and 24 ± 7 °C, respectively, and relative humidity was 55 ± 7%, 51 ± 10% and 54 ± 14% (Table 1).

**Table 1:** Resting values in the separate exercise conditions

Resting values (10 min)	Land	32 °C	38 °C
MCAv (cm/s)	57 ± 10*	61 ± 10	56 ± 8*
MCA CVC (cm.s/mmHg)	0.647 ± 0.120	0.694 ± 0.104	0.646 ± 0.244
PETCO_2_ (mmHg)	24 ± 6	26 ± 5	27 ± 6
Mean arterial pressure (mmHg)	88 ± 8#	88 ± 11#	79 ± 8
Core temperature (°C)	37.17 ± 0.58	36.9 ± 0.40	37.09 ± 0.21
Heart rate (bpm)	72 ± 11*	64 ± 12	73 ± 11*
VO_2_ (ml/kg/min)	4.5 ± 0.5	4.2 ± 1.0	4.3 ± 0.9
Brachial blood flow (ml/min)	82 ± 81	66 ± 89	134 ± 109*

Values are means ± SD

*MCAv* middle cerebral artery velocity, *MCA CVC* middle cerebral artery cerebrovascular conductance.

Statistical significance was set at *P* < 0.05. *denotes significant difference from 32 °C**.** ^#^denotes significant difference from 38 °C

### Experimental measures

#### Cerebral and brachial blood flow

A 2-MHz ST3 Transcranial Doppler (TCD) ultrasound system (Spencer Technologies, Seattle, WA) was used to measure middle cerebral artery velocity (MCAv). The MCAv signal was identified via examination of velocity, waveform and depth as comprehensively described elsewhere (Aaslid et al. 1982). MCAv was collated via PowerLab exported in raw analogue form to LabChart (LabChart 8; ADInstruments, Sydney, Australia). MCAv conductance (CVC) was calculated as MCAv/mean arterial pressure (MAP). Brachial artery diameter and velocity were measured using high-resolution ultrasound (T3300, Terason, Burlington, MA) and analysed using custom-designed edge detection and wall-tracking software as described previously (Woodman et al. 2001). Mean values of the 5-min intervals prior to each time point were subsequently calculated.

#### Core temperature

Core temperature was measured using the wireless CorTemp core body temperature monitoring system (CorTemp, HQInq, Palmetto, FL, USA). Subjects ingested a CorTemp temperature sensor telemetry capsule ~ 6–7 h prior to experiment onset to ensure the sensor was at an ideal point in digestive tract during data collection. Readings were taken every 5 min with the hand-held CorTemp data monitor.

#### Respiratory measures

Oxygen consumption (VO_2_) and end-tidal carbon dioxide (PETCO_2_) were recorded via Parvo Medics TrueOne® metabolic cart (Parvo Medics, Salt Lake City, UT, USA) with associated mouthpiece and tubing. One-way valves and a nose peg were utilised to ensure end-tidal gases were accurately analysed. Mean values from the 5-min period prior to time points were averaged.

#### Systemic haemodynamics

Blood pressure was continuously recorded via photo plethysmography using a Finometer finger cuff (Finometer Pro, Finapres Medical systems, The Netherlands) and exported continuously to PowerLab throughout the experiment. Subjects placed their left arm on a platform at approximately heart level whilst a height sensor was taped to the torso at atrium level to automatically account for elevation changes of the finger cuff. Mean arterial pressure and heart rate (HR) were calculated in real time by PowerLab cyclically using the formula (1/3 SBP + 2/3 DBP) and measuring systolic peak rate, respectively.

### Statistics

SPSS 23.0 (SPSS, Inc., Chicago, IL) was used for statistical analysis. Two-way repeated-measures ANOVAs were performed to compare conditions in addition to across timepoints. Post hoc comparisons between paired data points were undertaken using paired *t* tests with LSD correction. Statistical significance was set at *P* < 0.05. All data are mean ± standard deviation, unless stated otherwise.

## Results

### Cerebrovascular and respiratory variables

**Fig. 1:**
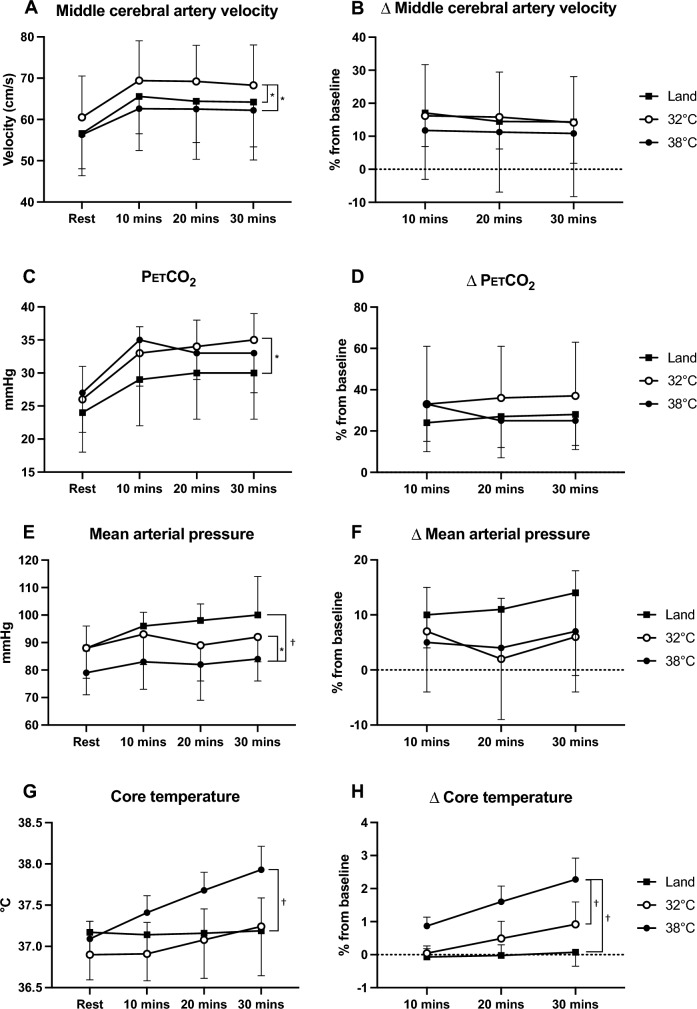
Middle cerebral artery velocity (MCAv, panel **A,** **B**), partial pressure of end-tidal carbon dioxide (PETCO_2_, panel **C**, **D**), mean arterial pressure (panel **E**, **F**) and core temperature (panel **G**, **H**) at rest and throughout the 30 min exercise bout in the three separate conditions. Data presented as mean ± standard deviation, with (*) indicating a significant difference between conditions at *P* < 0.05, and (†) a significant difference at *P* < 0.001 derived from a two-way repeated-measures ANOVA

**Fig. 2:**
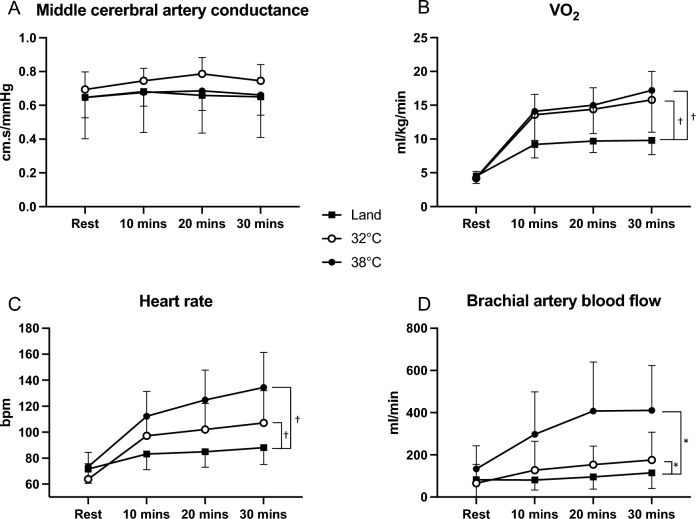
Middle cerebral artery conductance (MCA CVC, panel **A**), volume of oxygen consumption (VO_2_, panel **B**), heart rate (panel **C**) and brachial blood flow (panel **D**) at rest and throughout the 30 min exercise bout in the three separate conditions. Data presented as mean ± standard deviation, with (*) indicating a significant difference between conditions at *P* < 0.05, and (†) a significant difference at *P* < 0.001 derived from a two-way repeated-measures ANOVA

Whilst MCAv increased significantly from the onset of exercise in all conditions (*P* < 0.001), MCAv was significantly higher at rest in the 32 °C condition compared to the Land (61 ± 10 vs 57 ± 10 cm/s, *P* = 0.02) and 38 °C conditions (56 ± 8, *P* = 0.03, Fig. 1A, B). Similarly, MCAv remained higher throughout the 32 °C exercise bout compared to the Land and 38 °C conditions (*P* = 0.03 and *P* = 0.02, respectively). No statistical difference was evident in MCA CVC between the conditions (Fig. 2A, *P*  = 0.30).

PETCO_2_ increased during exercise in all conditions (*P* < 0.001), with a significant interaction effect revealing PETCO_2_ was lower in the Land compared to the 32 °C condition (*P* = 0.02), whilst there was no difference between 32 °C and 38 °C immersion exercise (*P* = 0.99, Fig. 1C, D). No difference was observed in VO_2_ at rest between conditions, whilst exercise increased VO_2_ in all conditions (*P* < 0.001). Post hoc analysis revealed VO_2_ increased similarly in the 32 °C and 38 °C exercise bouts, and these were significantly different compared to the Land condition (Fig. 2B, both *P* < 0.001).

### Systemic haemodynamic and core temperature variables

MAP responses were different between conditions (main effects for condition and time, *P* = 0.005 and *P* = 0.001, respectively), with MAP significantly lower at rest and throughout the 38 °C exercise bout compared to the Land (*P* < 0.001) and 32 °C conditions (*P* = 0.03, Fig. 1E, F). No difference was observed between the 32 °C and Land responses (*P* = 0.11). HR increased in a stepwise manner throughout exercise in all conditions (interaction effect *P* < 0.001), with the greatest increase observed in the 38 °C condition compared to the 32 °C and Land conditions (Fig. 2C, both *P* < 0.001).

There was a significant interaction effect for core temperature (*P* < 0.001), whereby no differences were observed in the Land and 32 °C exercise bouts at rest or in response to exercise, whilst core temperature increased 0.84 ± 0.24 °C during the 38 °C condition, and was different from the 32 °C condition (Fig. 1G, H, *P* < 0.001). Consistent with the core temperature and subsequent thermoregulatory response, there was an interaction effect for brachial blood flow (*P* < 0.001), where it was highest in the 38 °C condition, and significantly different from the Land (*P* = 0.003) and 32 °C bouts (*P* = 0.006, Fig. 2D).

## Discussion

We examined the impact of water immersion and temperature on CBF velocity during aqua-cycling in healthy young subjects. Our findings extend our previous observations, and reinforce other studies (Carter et al. 2014; Pugh et al. 2014; Parfitt et al. 2017; Shoemaker et al. 2019), by indicating that cycle exercise in 32 °C water induces an increase in CBF velocity in humans. Our novel findings indicate that cycle exercise in water at 38 °C attenuates the beneficial impact of 32 °C water immersion on CBF velocity, due to redistribution of blood to peripheral vascular beds to subserve thermoregulatory heat loss.

Our study was based on evidence that improving cerebrovascular function is linked to clinical benefit, in terms of decreasing stroke and dementia risk. The burden of these diseases is very large and has been increasing in recent decades (Feigin et al. 2021). Designing optimal exercise interventions to prevent the progression of these diseases is, therefore, an important agenda in clinical exercise physiology, but it relies on understanding the mechanisms responsible for the benefits of exercise. Whilst there is strong epidemiological evidence relating fitness and activity levels to cerebrovascular outcomes (Hillman et al. 2008; Gallanagh et al. 2011), the mechanisms linking exercise and brain health remain largely unknown. Based on studies in peripheral (Green et al. 2017) and coronary (Hambrecht et al. 2003) arteries linking repetitive shear stress to improvements in endothelial function, we have proposed that exercise may induce benefits for brain health via impacts on endothelial function. To test this hypothesis, we recently undertook a 6-month intervention study comparing water- (30 °C) and land-based walking in older healthy subjects and found that water-based exercise induced changes in cerebral autoregulation (Green et al. 2021). Optimising the exercise stimulus, in this case the impact of exercise on cerebral blood flow and shear stress, may enhance cerebrovascular benefit, and the current study was designed to address the impact of water temperature in this regard. Our findings indicate that water temperature may be a key determinant of vascular benefit, given that the 38 °C condition was not associated with increases in CBF velocity during cycle exercise relative to the 32 °C condition.

Our current study provides some integrative physiology insights. CBF is driven by a number of factors, including cerebral metabolism, blood CO_2_ levels, and blood pressure. In the current study, CBF velocity was higher in the 32 °C condition than the Land condition, despite comparable BP responses to cycling. In our previous study comparing the acute effects of walking on land and in the water (Pugh et al. 2014), we observed higher BP responses during water immersion, suggesting that the hydrostatic impact of immersion centralises blood volume and, via a Starling effect, enhances cardiac output. In the current experiment involving cycle exercise, BP was not higher during immersion, possibly indicating that walking and cycling modalities of exercise may differ. It is equally pertinent that the level of immersion differed between this cycle study (immersion to the umbilicus) and our previous walking experiment (immersion to the right atrium level) (Carter et al. 2014; Pugh et al. 2014). In any event, CBF velocity was higher in this study during 32 °C immersion to the hip than the Land condition. PETCO_2_ was also higher in the 32 °C condition than Land and this may explain, at least in part, the elevated CBF associated with water immersion. It is important to note that we do not have direct measures of cerebrovascular metabolism, although no differences were reported in perceived effort between the Land and 32 °C conditions.

Comparison of the 38 °C and 32 °C conditions also provides some mechanistic insight. The CBF velocity responses were lower in 38 °C which may be attributable to the lower BP responses across the exercise bout. It appears that increased core temperature drives peripheral vasodilation to subserve thermoregulatory heat loss, supported by our findings of increased upper limb blood flow responses in the 38 °C condition. Whilst previous studies have indicated that higher body temperatures are associated with increased brain metabolism (Bain et al. 2020), our observation of lower CBF velocity in the warmer condition suggests that thermoregulatory drive is paramount in the integrative response to immersion, and exercise in warmer water. However, although not statistically different, PETCO_2_ was somewhat lower in the 38 °C condition than 32 °C at the 20 and 30 min timepoints. This may be the result of hyperthermia-induced hyperventilation (Nybo and Nielsen 2001), and given that the cerebral circulation is highly sensitive to small changes in arterial CO_2_ blood gas levels, it is possible that this was a factor that attenuated the CBF response at 38 °C. Taken together, our findings suggest a hierarchy of mechanistic control of CBF velocity during water immersion and heat exposure. When heat dissipation is not an issue (i.e. comparing 32 °C and Land condition), PETCO_2_ appears to be an important driver of CBF velocity responses. However, when core temperature rises and systematic haemodynamic and respiratory function changes to regulate body temperature (as evidenced by comparing 38 °C and 32 °C), PETCO_2_ in addition to blood pressure appears to collectively determine CBF.

There are several limitations to this study. We did not include cold temperature conditions and this may be an interesting topic for future experiments. Future studies may also modify water levels to address whether hydrostatic effects play a greater role during immersion to the right atrial level. Our measure of cerebral vascular responses relied on transcranial Doppler ultrasound, which has some acknowledged shortcomings when deriving blood flow outcomes due to the lack of information regarding arterial diameter changes in the brain. Finally, we were unable to obtain reliable echocardiographic measures of stroke volume during cycle exercise in this experiment, which limits our capacity to assume changes in venous return and cardiac output.

## Conclusion

Our study confirms previous experiments regarding the impact of 32 °C water immersion on cerebrovascular responses and extends this observation to a beneficial impact during aquatic cycle ergometry. It is also the first study to address the impact of water temperature on integrative physiological responses and brain blood flows. We conclude that whilst water-based exercise can have beneficial effects on cerebrovascular function, water temperature is a key modulator of this benefit.

## Data Availability

The data underlying this article will be shared upon reasonable request to the corresponding author.
